# An Improved Sequencing-Based Bioinformatics Pipeline to Track the Distribution and Clonal Architecture of Proviral Integration Sites

**DOI:** 10.3389/fmicb.2020.587306

**Published:** 2020-10-20

**Authors:** Nicolas Rosewick, Vincent Hahaut, Keith Durkin, Maria Artesi, Snehal Karpe, Jérôme Wayet, Philip Griebel, Natasa Arsic, Ambroise Marçais, Olivier Hermine, Arsène Burny, Michel Georges, Anne Van den Broeke

**Affiliations:** ^1^ Laboratory of Experimental Hematology, Institut Jules Bordet, Université Libre de Bruxelles (ULB), Brussels, Belgium; ^2^ Unit of Animal Genomics, GIGA, Université de Liège (ULiège), Liège, Belgium; ^3^ VIDO-Intervac, University of Saskatchewan, Saskatoon, SK, Canada; ^4^ Service d’hématologie, Hôpital Universitaire Necker, Université René Descartes, Assistance publique hôpitaux de Paris, Paris, France; ^5^ Institut Imagine, INSERM U1163, CNRS ERL8654, Paris, France

**Keywords:** human T-cell leukemia virus type-1, bovine leukemia virus, linear amplification-mediated-PCR, adult T-cell leukemia, retrovirus, clonal architecture, bioinformatics pipeline, integration site

## Abstract

The combined application of linear amplification-mediated PCR (LAM-PCR) protocols with next-generation sequencing (NGS) has had a large impact on our understanding of retroviral pathogenesis. Previously, considerable effort has been expended to optimize NGS methods to explore the genome-wide distribution of proviral integration sites and the clonal architecture of clinically important retroviruses like human T-cell leukemia virus type-1 (HTLV-1). Once sequencing data are generated, the application of rigorous bioinformatics analysis is central to the biological interpretation of the data. To better exploit the potential information available through these methods, we developed an optimized bioinformatics pipeline to analyze NGS clonality datasets. We found that short-read aligners, specifically designed to manage NGS datasets, provide increased speed, significantly reducing processing time and decreasing the computational burden. This is achieved while also accounting for sequencing base quality. We demonstrate the utility of an additional trimming step in the workflow, which adjusts for the number of reads supporting each insertion site. In addition, we developed a recall procedure to reduce bias associated with proviral integration within low complexity regions of the genome, providing a more accurate estimation of clone abundance. Finally, we recommend the application of a “clean-and-recover” step to clonality datasets generated from large cohorts and longitudinal studies. In summary, we report an optimized bioinformatics workflow for NGS clonality analysis and describe a new set of steps to guide the computational process. We demonstrate that the application of this protocol to the analysis of HTLV-1 and bovine leukemia virus (BLV) clonality datasets improves the quality of data processing and provides a more accurate definition of the clonal landscape in infected individuals. The optimized workflow and analysis recommendations can be implemented in the majority of bioinformatics pipelines developed to analyze LAM-PCR-based NGS clonality datasets.

## Introduction

A hallmark of all retroviral infections and an essential step in the life cycle is the proviral DNA integration into the host genome (Demeulemeester et al., 2015). More than a lifelong signature, the clonal distribution of proviral integration sites (IS) significantly influences the fate of infected cells (Cohn et al., 2015; Rosewick et al., 2017). Recently, the application of linear amplification-mediated PCR (Schmidt et al., 2002, 2007; LAM-PCR) and next-generation sequencing (NGS) has facilitated the identification of hundreds of thousands of retroviral integration sites genome-wide while simultaneously measuring the abundance of the corresponding clones (Paruzynski et al., 2010; Gillet et al., 2011; Firouzi et al., 2014; Maldarelli et al., 2014; Wagner et al., 2014; Sunshine et al., 2016; Artesi et al., 2017; Rosewick et al., 2017). Analyses by us and others have demonstrated that the integration sites of human T-cell leukemia virus type-1 (HTLV-1; Gillet et al., 2011; Melamed et al., 2013; Cook et al., 2014; Rosewick et al., 2017), bovine leukemia virus (BLV; Gillet et al., 2013; Rosewick et al., 2017), avian leukemia virus (ALV; Justice et al., 2015; Malhotra et al., 2017), and human immunodeficiency virus-1 (HIV-1; Maldarelli et al., 2014; Wagner et al., 2014; Cohn et al., 2015; Einkauf et al., 2019) are not random and, in the case of BLV/HTLV-1, revealed hotspots of proviral integration sites in the vicinity of cancer driver genes (Rosewick et al., 2017). The application of NGS clonality approaches to HIV-1 seropositive patients treated with combination antiretroviral therapy (cART) uncovered viral reservoirs of latently infected cells responsible for rapid viral rebound if the therapy is interrupted (Finzi et al., 1999; Maldarelli et al., 2014). Analysis of HIV-1 clonality also revealed subsets of clonally expanded cells often containing defective proviruses, although intact infectious HIV-1 has also been reported in highly expanded clones (Cohn et al., 2015; Simonetti et al., 2016). In addition to natural retroviral infections by exogenous and endogenous retroviruses, transduction strategies utilizing retroviral vectors have been developed and widely used for gene therapy applications (Cavazzana-Calvo et al., 2000). Potential adverse effects resulting from insertional mutagenesis caused by retro‐ and lentiviral vector-mediated transduction of target cells can be tracked by NGS-based mapping of vector integration sites (De Ravin et al., 2016). Systematic exploration of insertion sites is currently recommended by the FDA as an approach to assess the risk of integration-related effects in gene therapy clinical trials (FDA Center for Biologics Evaluation and Research, 2019).

**Figure 1:**
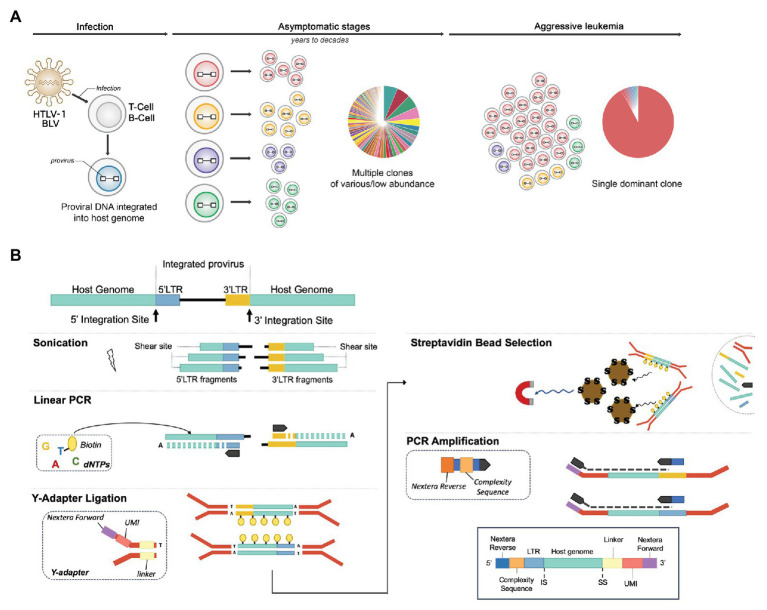
NGS clonality assays to explore the clonal landscape of infected cells. **(A)** Integration site distribution and clonal landscape in human T-cell leukemia virus type-1 (HTLV-1) and bovine leukemia virus (BLV)-associated malignancies. HTLV-1 and BLV infect T cells and B cells respectively, producing a chronic infection in their respective hosts (human and cattle/sheep) that evolves into full-blown leukemia/lymphoma in ~5% of individuals. Infected cells carry the viral genetic material or “proviral” DNA stably integrated into their genome. Early and chronic stages of infection are characterized by a large number of clones of low abundance, each uniquely identified by their proviral integration site in the genome. In each individual, the virus persists in several thousand distinct infected cell clones. Following a protracted latency period, for unknown reasons, one of these clones dramatically expands, leading to the accumulation of malignant cells in the blood (leukemia) or peripheral tissues (lymphoma). Exploring the clonal landscape over time is critical in understanding tumor evolution. Next-generation sequencing (NGS) clonality assays have the capacity to identify proviral integration sites genome-wide and simultaneously measure the abundance of the corresponding clones. **(B)** NGS library preparation. DNA is sheared by ultrasonication and fragments of 300–700 bp are further processed by linear extension with biotinylated dTTPs and long terminal repeat (LTR)-specific primers (5'‐ and 3'-specific). A ligation step adds an adapter, providing a random tag, a linker, and Nextera forward sequences to the fragments containing LTR-host junctions. After capture with streptavidin beads, DNA fragments are PCR amplified, which adds the Nextera reverse sequences and a stretch of random nucleotides to produce sequence complexity. The products are then amplified using off-the-shelf Nextera indexes to enable multiplexing. Libraries are sequenced on a MiSeq instrument (kit V2, 2 × 150 bp).

While NGS techniques based on capture probes have generated a genome-wide picture of HTLV-1 and HIV-1 integration sites and their associated proviral genomes (Iwase et al., 2019; Katsuya et al., 2019), capture-dependent approaches are limited in their throughput and are not well suited to exploring early nonmalignant stages of infection where only a small fraction of the cells carries the integrated provirus. We and others have used more high-throughput NGS integration site profiling approaches consisting of LAM-PCR followed by NGS (Paruzynski et al., 2010; Gillet et al., 2011; Firouzi et al., 2014; Sunshine et al., 2016; Percher et al., 2017; Rosewick et al., 2017). These methods share a first step of DNA fragmentation by restriction enzyme digestion or ultrasonic shearing of the genomic DNA followed by viral long terminal repeat (LTR)-specific amplification of the adjacent genomic sequences. The amplification product spans the virus–host junction, revealing the integration site. Libraries are produced by PCR amplification of adapter-ligated products. Three NGS methods have been developed to specifically explore the clonal architecture of virus-infected cells in HTLV-1‐ and BLV-infected individuals (Gillet et al., 2011; Firouzi et al., 2014; Rosewick et al., 2017). In the method described by Gillet (2011), sheared DNA fragments are end-repaired and tailed with a 3' A-overhang before ligating a Y-shaped adapter. Fragments are PCR amplified with primers located in the 3' LTR and the adapter region. Combined information regarding the integration site and the shear site defines clone abundance. Work by Firouzi and colleagues (Firouzi et al., 2014) improved the estimation of clonal abundance by the introduction of unique molecular identifiers (UMI). Amplification is performed by nested-splinkerette PCR, targeting the 3' LTR (Potter and Luo, 2010). In the method developed in our laboratory (Rosewick et al., 2017; Figure 1), the sheared DNA fragments are selectively end repaired and A tailed *via* Taq polymerase extension from LTR-specific primers that extend into the host DNA from both the 5' and 3' LTRs. Biotinylated dTTPs are also incorporated during this extension step. Y-shaped adapters containing UMIs are ligated to the A overhangs, and streptavidin bead-mediated selection is carried out, followed by PCR amplification. Removal of PCR duplicates combining UMI, shear site, and integration site information ensures accurate estimation of clone abundance. Overall, our approach comprises several modifications compared to the previously reported protocols. The modified method assesses both the 5' and 3' LTR flanking sequences, increases the sensitivity, and significantly reduces the cost, making it a viable technique for application in the clinic. In a longitudinal study of adult T-cell leukemia (ATL) patients, we demonstrated that the optimized NGS mapping protocol applied to HTLV-1 outperformed other currently available methods, enabling the detection of patients refractory to first-line therapy and providing a better estimation of response to therapy (Artesi et al., 2017). We have then applied this method to samples collected from HTLV-1‐ and BLV-infected individuals of human, ovine, and bovine origin as well as HTLV-1 infected humanized mice, reflecting both basic and clinical settings (Artesi et al., 2017; Percher et al., 2017; Rosewick et al., 2017; Pérès et al., 2018; Galli et al., 2019; Marçais et al., 2020). This generated an unprecedented volume of raw sequencing data. Here, we report the bioinformatics workflow and describe critical modifications in the pipeline, improving processing speed, increasing both the specificity and the sensitivity of the assay, and reducing bias associated with proviral integration within low complexity regions of the genome. We provide an updated data analysis protocol and recommendations applicable to the majority of bioinformatics pipelines developed to analyze LAM-PCR-based NGS clonality datasets.

## Materials and Methods

### Participants and Material

NGS clonality datasets were obtained from peripheral blood mononuclear cells collected from ATL patients (HTLV-1, human dataset) and BLV-infected animals (asymptomatic and tumor stages) described in Artesi et al. (2017) and Rosewick et al. (2017). The human studies were approved by the ethics committee CPP Ile de France II, CNIL: number 1692254 and registration number 000001072. Animal experimental procedures were approved by the University of Saskatchewan Animal Care Committee, following the Canada Council on Animal Care Guidelines.

### Library Preparation and Sequencing

Clonality libraries were prepared according to Rosewick et al. (2017) and Artesi et al. (2017; Figure 1B). Briefly, DNA is sheared, and fragments of 300–700 bp are selectively end repaired and A tailed with biotinylated dTTPs and LTR-specific primers. Fragments containing LTR–host junctions with ligated adaptors providing a random tag (for PCR duplicate identification) and a linker (multiple linkers are used to identify cross-contamination) are then captured with streptavidin beads. DNA fragments are PCR amplified, and after purification of the products, a second PCR is performed, which adds off-the-shelf Nextera indexes. Libraries are sequenced on a MiSeq instrument (kit V2, 2 × 150 bp).

### Proviral Integration Site Calling

A detailed step-by-step description of the proviral integration site calling pipeline is available on github: https://github.com/GIGA-AnimalGenomics-BLV/Public/tree/master/PIC.

### Testing Blast Versus Bowtie2

Bowtie2 v2.2.9 index (bowtie2-build) and BLAST v2.6 (makeblastdb-dbtype nucl) databases were constructed from the BLV_YR2 3'LTR (8,694–8,720 bp, BLV YR2, Genbank: KT122858.1) or HTLV_ATK (9011–9,055 bp, GenBank: J02029) sequence. Ten million reads containing either the BLV or HTLV-1 3'LTR sequence were randomly selected from our NGS clonality sequencing dataset. Datasets were downsampled to different sets of various read numbers with seqtk.^1^ FASTQ files were transformed into FASTA with fastq_to_fasta (-Q33 -r -n) keeping only bases with Phred score > 33.^2^ FASTA sequences were compared to the BLAST database using blastn -word_size 7 -evalue 1. FASTQ sequences were aligned with bowtie2 (-L 14 -N 1 -U). Homemade R scripts were used to compare the outputs.

### Impact of Read Trimming on Proviral Integration Site Mapping

Bowtie2 indexes were constructed with BLV LTRs or HTLV-1 LTRs as separate chromosomes (BLV, LTR5: 1–30 bp, LTR3: 8,694–8,720 bp; HTLV, LTR5: 1–29 bp, LTR3: 9,011–9,055 bp). Reads from BLV-infected sheep (*n* = 516) or HTLV-1-infected individuals (*n* = 187) containing the appropriate linker sequence were mapped to their respective index with bowtie2 -L 14 -N 1 -U prior to or after trimming. Trimming was performed in two steps. First, the read section expected to contain the viral LTR was isolated with fastx_trimmer. Next, cutadapt -n 2 -m 10 was used to remove residual sequences, which arise from read overlap (see github for details). Custom R scripts were used to compare the outputs.

### Integration Site Recall

A recall procedure was performed using custom R scripts [available in the github R package PIC function getISposition (…, RECALL = TRUE)]. Trimmed reads from 184 HTLV-1 infected samples with at least 300 reads after removal of PCR duplicates were mapped with bowtie2 -very-sensitive -N 1 to the combined human genome (hg19) with the HTLV-1 proviral genome as an additional chromosome (HTLV_ATK, GenBank: J02029). Using the R package PIC, the position and abundance of integration sites were first called with high-confidence reads mapped in proper pairs (SAM flags 83, 99, 147, 163, and MAPQ ≥ 33). For each integration site, the RECALL abundance was calculated from the set of reads located in a window spanning 600 bp up‐ and downstream of the high-confidence integration site. Multi-mapped reads attributed to two distinct integration sites were assigned using clone abundance as weight factor. Like with high-confidence reads, PCR duplicates were removed based on the integration site, shear site, and random tag information.

### Clean and Recover Step

Integration sites (704,485) were acquired from longitudinal samples collected from 30 BLV-infected sheep over >5 years (*n* = 553, unpublished data). From this dataset 377,710 unique integration sites were obtained. Integration sites found in only one animal were retained, while those detected in >1 animal were further explored. Their relative distribution across all serial samples of each individual was assessed by computing the Shannon entropy (Shannon, 1948) of their recurrence (= number of times this integration site is detected in each animal; S_r_) and highest abundance across all animals and all samples (S_a_). Integration sites with a S_r_ or S_a_ <0.85 were attributed to the animal with the highest recurrence (if S_r_ <0.85) or the highest abundance (if S_a_ <0.85) of that particular integration site. Integration sites that did not meet any of these criteria were flagged as “uncertain” and excluded, while the others were retrieved. This category contains integration sites found at a similar occurrence in different animals and at a low abundance in each of them. A detailed description of the protocol is available on github https://github.com/GIGA-AnimalGenomics-BLV/Public/tree/master/PIC/R. The corresponding function is called tagContamination().

### Availability of Data and Materials

Integration site information and NGS clonality datasets are available from previous publications (Artesi et al., 2017; Rosewick et al., 2017). The datasets used and analyzed during the current study are also available from the corresponding author on request. The full code and a detailed step-by-step description of the bioinformatics pipeline is available at https://github.com/GIGA-AnimalGenomics-BLV/Public/tree/master/PIC.

## Results

### Bioinformatics Workflow

**Figure 2:**
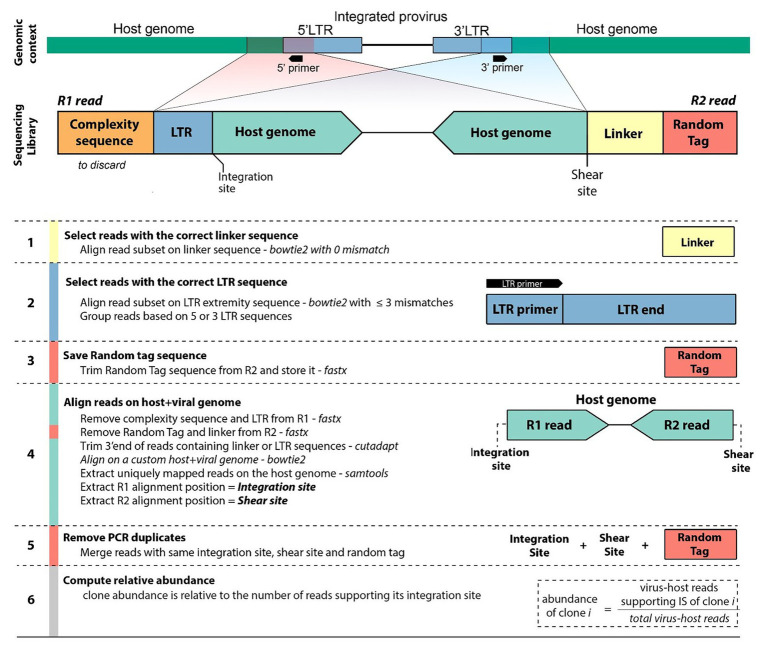
Workflow diagram. Sequencing of clonality libraries produces pairs of 150-bp reads. Read 1 (R1) contains a complexity sequence, a partial LTR sequence from the LTR primer to the LTR extremity (LTR), and the flanking host genomic sequence defining the integration site. Read 2 (R2) contains a random tag, the linker sequence, and the host genomic sequence defining the shear site. Read pairs originate from either the 5'LTR or the 3'LTR (shown here is the 3'LTR configuration). The pipeline identifies the integration site, shear site, and random tag of each pair of reads (see Materials and Methods section). Reads sharing the same integration site are grouped and, the abundance of the corresponding clone is computed after removal of PCR duplicates.

Libraries were prepared according to Rosewick et al. (2017) and Artesi et al. (2017), and sequenced on an Illumina MiSeq instrument with 2 × 150-bp reads (see Materials and Methods section). Read 1 (R1) consists of three elements. First a stretch of six random nucleotides (complexity sequence), which provide higher sequence diversity at the 5' read extremity and improve sequencing quality on the Illumina platform. Second, a proviral LTR sequence consisting of the primer sequence used for linear amplification followed by the downstream LTR extremity (LTR). Third, the flanking host genomic sequence corresponding to the integration site. Read 2 (R2) contains an 8-bp random tag ligated prior to PCR amplification, a 14-bp linker sequence unique to each library to avoid cross-contamination between libraries, followed by the host genomic sequence defining the random shear site generated by sonication. We designed a six-step workflow to analyze the NGS datasets (Figure 2). The full code of the bioinformatics pipeline is available at https://github.com/GIGA-AnimalGenomics-BLV/Public/tree/master/PIC.

### Accelerating Data Processing: Favoring Short-Read Aligners Over BLAST/BLAT

**Figure 3:**
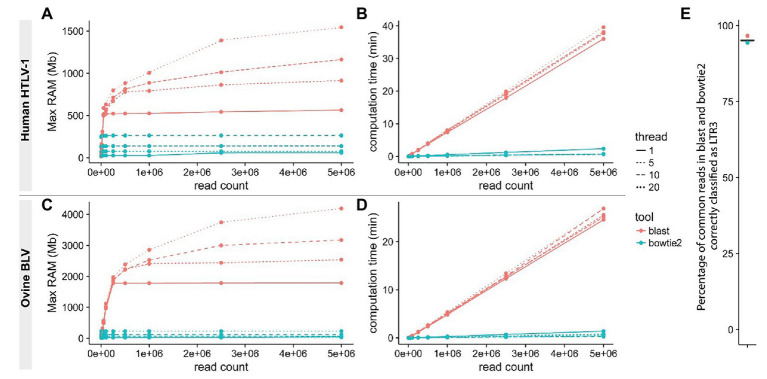
Improving read mapping processing speed. Reads from BLV **(A,B)** or HTLV-1 **(C,D)** clonality libraries containing 3'LTR-specific sequences were identified using either bowtie2 (-L 14 -N 1 -U; blue) or BLAST (red) with variable thread numbers (line types). RAM usage **(A,C)** and speed **(B,D)** were examined. The proportion of reads containing the appropriate 3'LTR sequence was compared between the two procedures **(E)**.

Reducing computational time becomes a priority when analyzing large volumes of data. Recently reported pipelines primarily use traditional local alignment tools like BLAST/BLAT (Altschul et al., 1990; Kent, 2002) for at least one step of the NGS clonality workflow. These tools perform poorly in protocols that typically require the alignment of several million short sequences (Paruzynski et al., 2010; Firouzi et al., 2014; Maldarelli et al., 2014; Cohn et al., 2015; Aoki et al., 2016; Farmanbar et al., 2017). We applied the short-read aligner bowtie (Langmead et al., 2009) or bowtie2 (Langmead and Steven, 2013) at every step of the protocol to a set of reads from our HTLV-1 and BLV clonality datasets (Figure 2, steps 1–2–4, see Materials and Methods section) and demonstrated that this dramatically reduced both processing time and memory footprint (Figure 3). In addition, short-read aligners consider base quality, significantly increasing alignment specificity.

### Trimming Short Reads to Improve Alignment

**Figure 4:**
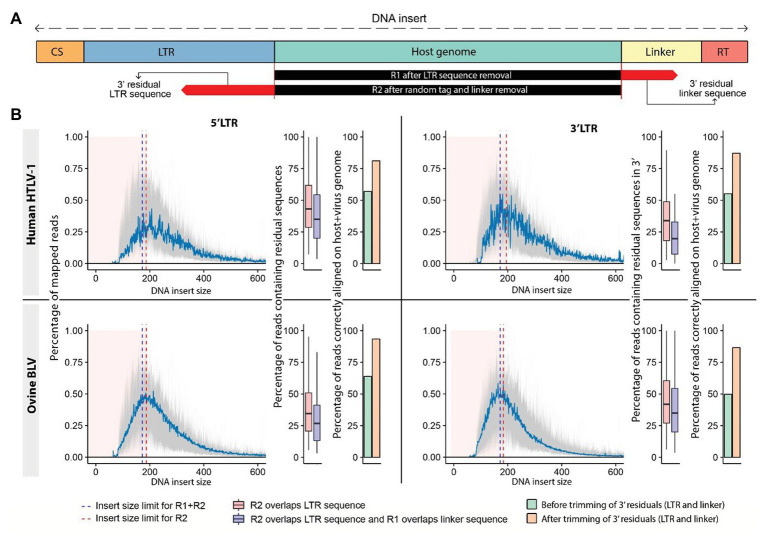
Trimming sequencing reads increases the number of reads supporting the proviral integration sites. **(A)** Schematic representation of sequenced DNA fragments. The DNA “insert size” is measured from the start to the end of the mapped portion of the DNA fragment, which corresponds to the host genomic sequence. R1 reads generated from small DNA inserts carry sequences corresponding to the linker of R2 at their extremity, while their R2 counterparts can contain a fraction of the LTR sequence found in R1 (residual sequences). **(B)** Distribution of insert sizes across mapped reads from HTLV-1 (human) and BLV (ovine) clonality libraries (mean represented in blue). Reads produced from short fragments affected by sequence overlap are shown by pink area. Overlap R2 (red dashed line), overlap both reads (blue dashed line). Boxplots: percentage of reads in each category. Barplots: percentage of reads mapping to the host genome prior to (green) and after (orange) trimming. 5' LTR data: left, 3' LTR data: right.

We observed that mapping of raw reads to the host genome resulted in a significant number of misaligned bases at the read extremities (soft-clipped sequences). The presence of mismatches increases the level of uncertainty in the alignment, which is reflected in the mapping quality score (MAPQ). Reads with a low MAPQ have a greater chance of being filtered out to avoid retaining misalignments. The majority of these mismatches result from the presence of sequencing adapters and viral sequences, which can be eliminated from reads prior to mapping them to a reference genome (Figure 4A). We used fastx_trimmer to remove adapters and proviral LTR sequences based on their respective theoretical length (Figure 2, step 3).^3^ In the majority of these cases, we observed a strong bias toward reads with short insert sizes (=distance between R1-start and R2-end alignment) characterized by frequent overlaps between reads R1 and R2 of the same pair (mean: 28.06%; Figure 4B). More specifically, we found that the sequence of one read of the pair frequently overlapped a region of its mate read, creating soft-clipped bases at the extremity of the fragment despite appropriate trimming of adapter and LTR sequences. The application of cutadapt (Martin, 2011) to remove the corresponding overlapping sequences significantly increased the number of uniquely mapped reads (up to 30%), resulting in a higher number of reads supporting the proviral integration site (Figure 4B). Bias toward small insert size is influenced by the fragment length distribution after ultrasonic shearing, PCR amplification, bead selection of amplified sequences, and library sequencing. The magnitude/severity of this bias may therefore vary among protocols.

### Adjusting the Accuracy of Clone Abundance Quantification for Integration Sites Located Within Repetitive Regions of the Host Genome

**Figure 5:**
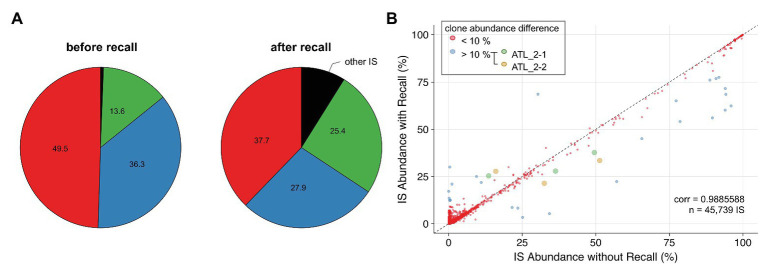
Applying a recall step improves accuracy of estimating clone abundance. **(A)** Pie charts representing proviral integration site distribution of ATL 2 generated with the conventional pipeline (left chart) or the recall procedure (right chart). Each slice of a pie represents an independent proviral integration site, and the size of the slice corresponds to the relative abundance (percentage in HTLV-1-infected cells). **(B)** Impact of the recall procedure on the clonality dataset (see Materials and Methods section) for integration sites supported by >15 reads. ATL_2-1 and ATL_2-2: two independent samplings of ATL 2.

Low-complexity sequences are frequent in eukaryotic genomes, and regions that contain such repetitive elements are difficult to resolve with short reads. Integration sites falling within low-complexity regions will often be attributed inaccurate clone abundances due to the poor mapping quality of sequencing reads supporting these sites. A representative example is ATL 2, a primary ATL classified as an acute leukemia subtype, which consists of a single dominant T-cell clone as revealed by the presence of a clonal T-cell receptor gamma gene rearrangement (Rosewick et al., 2017). Surprisingly, NGS mapping of proviral integration sites of ATL 2 revealed two major HTLV-1 insertions in chr5 and chr16 with a relative abundance of 49.5 and 36.3%, respectively, and a third minor integration site in chr1 representing 13.6% (Figure 5A). In contrast, the application of PCIP-seq, an Oxford Nanopore-based long-read sequencing method developed by us to capture proviral integration sites and simultaneously sequence the viral genome associated with each site (Artesi et al., 2019), revealed three major proviruses located on chr5, chr16, and chr1 in ATL 2, each responsible for 33.7, 34.5, and 31.8% of the HTLV-1/host hybrid reads, respectively. These data suggested the presence of three proviral integrations in a single T-cell clone. We observed that the proviral insertion site on chr1 falls within a repetitive element causing many of the short reads to map to multiple regions in the genome. As a result, most of the reads obtained with our short-read NGS clonality method had been discarded due to poor alignment quality (MAPQ < 30). In contrast to the long reads from PCIP-seq, which allow unambiguous mapping in repetitive regions, we observed that in our Illumina-based method, retaining multimapped reads produced values closer to reality (25.4%, Figure 5A, after recall). Therefore, to correct for the abundance of this integration site, we applied a two-pass “recall” step in our workflow. Integration sites were first defined according to the reads in proper pair (=SAM flag 0x2) with a high MAPQ > 30. Proper-pair reads are defined as mapped in pairs, in the expected relative orientation and within a distance compatible with the aligner’s parameters. In a second step, reads with lower mapping quality were recovered. For each integration site identified, we then recomputed the number of reads associated with that particular site using reads with lower mapping quality aligning to multiple positions located within a window spanning 600 bp up‐ and downstream of the integration site initially defined by the high MAPQ reads. Multimapped reads that matched several high confidence integration sites were preferentially attributed to the integration site with the highest abundance. Altogether, our findings suggest that applying an extra recall step in the clonality bioinformatics workflow results in a more accurate estimation of clone abundance for proviral integration sites localized in low-complexity repetitive regions of the genome. Applied to the entire NGS clonality dataset, the recall method did not dramatically affect the abundance of the majority of the clones (Figure 5B, cor = 0.9885588, *n* = 45,739).

### Cleaning Datasets and Recovering Integration Sites

We applied our high-throughput protocol to several hundred samples collected from HTLV-1‐ and BLV-infected individuals and after processing, the data obtained 35,758 and 302,740 unique proviral integration sites, respectively. This revealed recurrent integration sites across samples likely caused by incorrectly assigned reads as the chance of finding an identical insertion site in two independent individuals is extremely low. Possible causes are cross-contamination during library preparation, bioinformatics issues in the demultiplexing process or index hopping and carryover during or between sequencing runs, which have been described as the main source of misassignment when sample and library preparation are processed in rigorously controlled conditions. Addressing this concern is particularly critical in longitudinal studies, which basically aim at examining a cohort of infected individuals (HTLV-1 or BLV in our studies) as well as multiple serial samples from the same individual. Cross-contamination will result in the inaccurate identification of identical integration sites across distinct samples.

**Figure 6:**
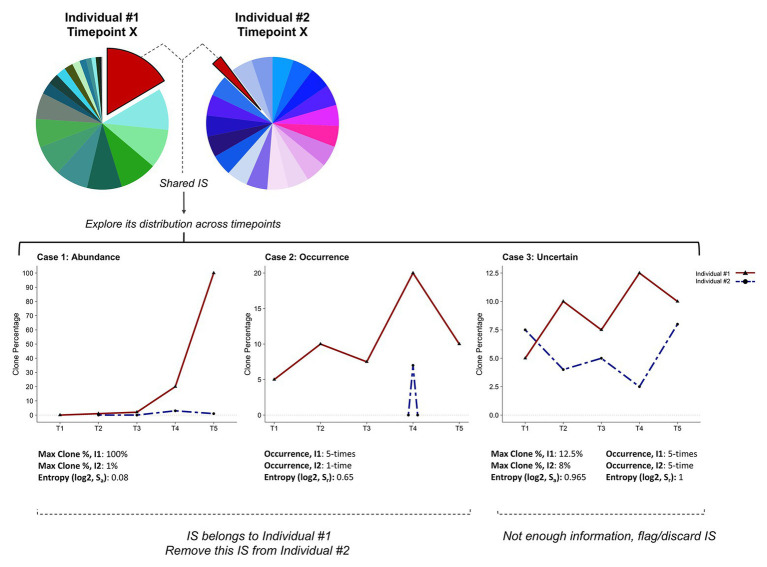
Cleaning clonality datasets and recovering integration sites. Schematic representation of the pipeline designed to address cross-contamination and carryover. The method is based on the assumption that analyzing the clone distribution among all time-points (i.e., T1–T5) and individuals (i.e., I1, I2) can discriminate real integration sites from cross-contamination/carryover. According to information on clone abundance and number of occurrences of a specific integration site in each individual, we define three categories. Case 1: the highest clone abundance across all samples is found in one individual (I1). Case 2: the integration site is recurrently found in a single individual (I1). Case 3: all individuals show similar clone abundances and integration site occurrences. Skewness in clone abundance and occurrence distributions is assessed by computing the Shannon entropy (S_a_ or S_r_). In Cases 1 and 2, the integration site is assigned to individual I1 and removed from individual I2’s integration site list. Integration sites that do not fulfill these criteria are flagged as uncertain and eliminated (Case 3).

To identify the sample to which the recurrent integration site should be assigned, we considered two parameters. First, the maximum number of reads associated with this particular integration site across all individuals and all samples (S_a_, “abundance”). Second, the maximum number of occurrences of this given integration site in each individual (S_r_, “recurrence”). For both parameters, we compute the Shannon entropy (Shannon, 1948), which recapitulates how skewed the presence is of an integration site in a given individual. Low Shannon entropy values indicate more diversity, with values close to zero representing the most dissimilar distributions. A schematic representation of the method is shown in Figure 6. Using empirical thresholds (here S_a_ or S_r_ <0.85), we assign each integration site to one of the following classes: abundance, occurrence, or uncertain. The “abundance” and “occurrence” categories define integration sites that can be attributed to a specific individual based on a higher proportion of reads identified in one individual (Figure 6, Case 1) or a greater recurrence of this insertion site in serial samplings collected from this individual (Figure 6, Case 2). Integration sites belonging to these categories will be assigned to a single individual and recovered. The third class defines integration sites that cannot be attributed to a unique individual with sufficient confidence (Figure 6, Case 3). IS classified as uncertain will be eliminated. Applied to our ovine BLV IS dataset (704,485 total IS, 377,710 unique IS prior to final merging), this approach resulted in the recovery of 23.75% (2,121 IS) of all recurrent integration sites (8,914 IS). This protocol appeared particularly useful for integration sites corresponding to abundant predominant clones, which are more likely to contaminate other samples.

## Discussion

The application of ligation-mediated PCR protocols combined with NGS is having a large impact on our understanding of retroviral pathogenesis. Recently, considerable effort has been expended to optimize NGS clonality methods and explore the clonal architecture of clinically important retroviruses like HTLV-1 and HIV-1. Once sequencing data are generated, the application of rigorous bioinformatics analysis is central to the biological interpretation of the data. To better exploit the potential of these methods, we developed an optimized bioinformatics pipeline for analyzing NGS clonality datasets. Here, we report a detailed workflow and describe a set of improved or additional steps in the computational protocol. We demonstrate that application of these steps to HTLV-1 and BLV clonality datasets provides a better estimation of proviral integration site distribution and a more accurate picture of the clonal landscape in infected individuals.

With the development of cost-effective NGS clonality methods and the availability of NGS platforms for routine diagnosis applications, there is increasing interest from the clinical community to monitor the clonal architecture of HTLV-1-infected ATL patients. Given the urgent need for novel therapeutic strategies for this poorly controlled T-cell malignancy, patients with aggressive and refractory forms of ATL will be increasingly enrolled in clinical trials requiring systematic monitoring of therapeutic responses. In addition to managing ATL patients, routine NGS clonality analysis of asymptomatic HTLV-1-infected individuals has the potential to identify carriers at greater risk of developing ATL. Such routine clinical applications require highly accurate measures of clone abundance. A short processing time for NGS data is a priority when answering questions regarding clinical outcomes for HTLV-1-infected patients. With the generation of massive volumes of sequencing data in the context of experimental studies, processing speed has also gained increasing importance in basic research. Here, we demonstrate that in contrast to BLAST/BLAT, short-read aligners specifically designed to manage NGS datasets offer increased speed, reducing processing times by an order of magnitude while accounting for sequencing base quality. In addition, short-aligner usage also lessens the computational burden that accompanies this step.

Both BLAST and most short-read aligners depend on seed-and-extend strategies (Li and Homer, 2010). If a sufficient match (or seed) between the sequencing read and the indexed reference genome is found, the alignment algorithm will attempt to extend it, assigning a different weight to a perfect match, a deletion, or a mismatch. Keeping mismatches or soft-clipped sequence stretches, which may stem from adapters or “residual” sequences from library preparation, critically impacts the alignment accuracy and the global output. We recommend a step to perfectly clean all sequencing reads from nongenomic contaminating sequences before mapping to the reference genome. Tools like cutadapt or fastx_trimmer have been developed to trim NGS reads, eliminating adapter and undesired sequences while also taking into consideration sequence quality. Our results demonstrate the utility of this additional cleaning step for adjusting the number of reads supporting an insertion site, providing a more accurate estimation of clone abundance.

To improve the accuracy of insertion site identification in repetitive regions of the host genome, we developed a recall procedure. Filtering low-quality NGS reads is highly recommended to avoid false positives; however, applied to low-complexity genomic regions, quality filters will impair the detection of proviral integration sites, inflating the abundance of integration sites assigned to higher-complexity genomic regions. We demonstrate that reads aligning to multiple regions in the genome can be used with a certain degree of confidence when a fraction of them map uniquely to that region. We further demonstrated that the recall procedure improved the accuracy of clone abundance estimates for HTLV-1 integration sites in repetitive regions of the human genome, enabling restoration of the clonal landscape in complex HTLV-1 samples.

Finally, we recommend the application of a “clean-and-recover” step especially for datasets generated from large cohorts and longitudinal studies. Applying this additional step to our dataset recovered about one fourth of the recurrent integration sites, which would otherwise have been eliminated. This set included integration sites corresponding to biologically important clones such as predominant malignant clones and their precursors. A major limitation of this approach is its dependence on empirical thresholds to discriminate between insertion sites that can be eliminated and those that should be retained. Integration sites supported by repeated occurrence within an individual and/or high abundance can be assigned with confidence and recovered; however, other contaminating integration sites should be assessed with caution and discarded.

## Conclusion

We further optimized the bioinformatics pipeline and describe critical steps in the computational processing protocol for NGS clonality datasets generated from HTLV-1 and BLV-infected individuals. We demonstrate that the application of rigorous steps in the bioinformatics process is critical to address some of the biases associated with the nature of LAM-PCR-based sequencing libraries. The application of our optimized bioinformatics protocol to HTLV-1 and BLV NGS clonality datasets decreases total execution time and reduces the bioinformatics burden while taking into account read quality. The workflow significantly improves the accuracy of estimating clone abundance by reducing biases associated with the presence of residual sequences and proviral integration within low-complexity regions of the genome. This updated workflow is applicable to the majority of bioinformatics pipelines developed to analyze NGS clonality datasets, requiring minimal adjustments. In addition to its application in research, it will be particularly useful for analyzing NGS clonality data from HTLV-1 patients when tracking risk to progression, disease evolution, and response to therapy.

## Data Availability Statement

Publicly available datasets were analyzed in this study. This data can be found here: European Nucleotide Archive (ENA) hosted by the European Bioinformatics Institute (EMBL-EBI) through study accession number PRJEB19394, Retroviral Integration Database (RID).

## Ethics Statement

The studies involving human participants were reviewed and approved by the ethics committee CPP Ile de France II, CNIL: number 1692254 and registration number 000001072. The patients/participants provided their written informed consent to participate in this study. The animal study was reviewed and approved by University of Saskatchewan Animal Care Committee following the Canada Council on Animal Care Guidelines, Protocol #19940212.

## Author Contributions

MA: prepared the libraries. KD and MA: developed the NGS protocol. NR and VH: were involved in data collection and developed the bioinformatics pipeline. NR, VH, KD, SK, JW, AB, MG, and AV: contributed to data analysis and interpretation. AM and OH: provided patient samples. PG and NA: collected animal samples. AV: supervised the research. NR, VH, and AV: wrote the manuscript. All authors contributed to the revision of the report and gave their final approval for submission.

### Conflict of Interest

The authors declare that the research was conducted in the absence of any commercial or financial relationships that could be construed as a potential conflict of interest.

## Footnotes

^1^https://github.com/lh3/seqtk

^2^http://hannonlab.cshl.edu/fastx_toolkit/

^3^http://hannonlab.cshl.edu/fastx_toolkit/

## Abbreviations

LAM-PCR: Linear amplification-mediated PCR
NGS: Next-generation sequencing
IS: Integration site
HTLV-1: Human T-cell leukemia virus-1
BLV: Bovine leukemia virus
ALV: Avian leukemia virus
HIV-1: Human immunodeficiency virus-1
cART: Combination antiretroviral therapy
LTR: Long terminal repeat
UMI: Unique molecular identifier
ATL: Adult T-cell leukemia
MAPQ: Mapping quality score
